# Dietary fiber fermentation restores microbiome resilience to oxygen stress in a host-independent *ex vivo* model

**DOI:** 10.1128/mbio.03934-25

**Published:** 2026-02-26

**Authors:** Yongjia Gong, Guojun Wu, Stephanie Tavarez, Cuiping Zhao, Liping Zhao, Shreya Ghosh

**Affiliations:** 1 Department of Food Science, School of Environmental and Biological Sciences, Rutgers, The State University of New Jersey 242612, New Brunswick, New Jersey, USA; 2 Center for Microbiome, Nutrition, and Health, New Jersey Institute for Food, Nutrition, and Health, Rutgers, The State University of New Jersey 242612, New Brunswick, New Jersey, USA; 3 Department of Microbiology and Biochemistry, School of Environmental and Biological Sciences, Rutgers, The State University of New Jersey 242612, New Brunswick, New Jersey, USA; University of California Irvine, Irvine, California, USA

**Keywords:** dietary fiber, oxygen stress, gut microbiota, Enterobacteriaceae

## Abstract

Gut dysbiosis is often driven by the loss of beneficial obligate anaerobes and the overgrowth of pathobiont facultative anaerobes, a shift perpetuated by oxygen buildup in the gut lumen. We hypothesized that dietary fibers could restore microbiome resilience to oxygen disruption by fueling short-chain fatty acid (SCFA) production and acidifying the environment to inhibit the blooming of pathobiont facultative anaerobes. To test this hypothesis independent of the host, fecal samples from healthy donors were cultured *ex vivo* under four conditions: Control, Oxygen-only (5%), Fiber-only, and Oxygen+Fiber. Guild-level analysis of 16S rRNA gene V4 region sequencing revealed that oxygen alone drove a significant expansion of co-abundant groups (CAGs; CAG2, CAG3) with facultative anaerobes, specifically amplicon sequence variants (ASVs) from Enterobacteriaceae. However, fiber supplementation under microaerophilic conditions fueled robust SCFA production and lowered pH, which effectively suppressed these oxygen-promoted pathobionts. Crucially, fiber restored the abundance of guilds (CAG15, CAG17) with key SCFA-producing obligate anaerobes, such as *Eubacterium* and *Roseburia*, which were otherwise inhibited by oxygen. Furthermore, we identified a unique guild (CAG19) containing *Bifidobacterium* that thrived specifically in the oxygen-fiber environment, suggesting a transitional role in restoring homeostasis. These findings demonstrate that dietary fibers can directly restore microbiome resilience against oxygen stress, offering a mechanistic strategy for managing dysbiosis-related conditions.

**IMPORTANCE:**

This study demonstrates that dietary fibers can protect short-chain fatty acid (SCFA)-producing obligate anaerobes under microaerophilic conditions in a host-independent manner. Unlike antioxidants that scavenge oxygen, fibers provide fermentable substrates that fuel microbial metabolism, lowering pH and creating a chemical environment that suppresses oxygen-tolerant pathobionts. Using an *ex vivo* fermentation model, we show that fiber drives the emergence of a *Bifidobacterium*-centered “transitional guild” that promotes the recovery of strict anaerobes, highlighting a microbe-fueled resilience mechanism. These findings reveal how dietary fibers directly stabilize the microbial community and support targeted high-fiber interventions to prevent dysbiosis.

## INTRODUCTION

Gut dysbiosis, characterized by an imbalance between beneficial obligate anaerobes and pathobiont facultative anaerobes, is a hallmark of chronic conditions such as inflammatory bowel disease (IBD) (1–3) and metabolic syndrome (MetS) (4, 5). While the prevalence of these chronic diseases has historically been highest in Western nations (6, 7), their incidence is rapidly rising in newly industrialized countries, paralleling urbanization and nutrition transitions (8, 9). A primary driver of this global health shift is the dramatic reduction in dietary fiber intake—plummeting from approximately 200 g per day in ancient populations to less than 20 g in modern industrialized societies (10, 11).

Dietary fibers are essential ecological drivers in the gut. They resist host digestion and serve as the primary energy source for the colonic microbiota (12). The fermentation of these fibers generates short-chain fatty acids (SCFAs), particularly butyrate, which is critical for maintaining the gut’s hypoxic niche. In a healthy gut, colonocytes oxidize butyrate to fuel their metabolism, a process that consumes significant oxygen and maintains a steep gradient from the oxygenated submucosa to the anoxic lumen (13, 14). This anoxia is crucial for the survival of obligate anaerobes. However, the “Oxygen Hypothesis” suggests that when fiber intake is low, butyrate production drops, causing colonocytes to shift metabolism and allowing oxygen to leak into the lumen (15–17). This oxygenation creates a selective advantage for facultative anaerobes—such as Enterobacteriaceae—allowing them to bloom and drive dysbiosis in a self-perpetuating feedback loop (16, 17).

While increasing dietary fiber is known to suppress these pathobionts and ameliorate inflammation (18–20), the precise mechanism remains debated. The prevailing view emphasizes a host-dependent pathway: fiber restores butyrate, which restores colonocyte hypoxia, which then suppresses facultative anaerobes (13). However, growing evidence suggests fiber may also exert a direct, host-independent protective effect by acidifying the luminal environment and producing antimicrobial SCFAs that chemically exclude oxygen-tolerant pathobionts (21, 22). To disentangle these mechanisms, we utilized an *ex vivo* fermentation model to test whether a specific high-fiber formula (23) can protect the gut microbiota from oxygen-induced dysbiosis in the complete absence of host regulation.

Finally, effectively capturing these ecological shifts requires moving beyond standard taxonomic analysis. Most microbiome studies group bacteria by phylogenetic relatedness (taxonomy). However, genomic diversity means that strains within the same species can exhibit vastly different functional responses to stress (24). For example, some strains of *Bifidobacterium* may be oxygen-tolerant while others are not. To overcome this limitation, we applied a guild-based analytical framework (25). By grouping bacteria based on co-abundance patterns—which reflect shared ecological behavior—rather than just their names, we aimed to identify the specific functional guilds that drive the restoration of microbiome resilience against oxygen stress (26).

In this study, we employed an *ex vivo* batch fermentation model to mimic gut microaerophilic stress (5% oxygen) in the presence or absence of a dietary fiber formula. By profiling the microbial community structure and metabolic output (SCFA and pH) across three healthy donors, we aimed to determine if fiber availability alone was sufficient to counter oxygen-induced dysbiosis. Using our guild-based analytical framework, we demonstrated that fiber fermentation created a “chemical shield” that effectively suppressed oxygen-tolerant pathobionts (such as Enterobacteriaceae) and rescued key SCFA-producing obligate anaerobes. Furthermore, we identified a unique “transitional guild” that thrived specifically in the oxygen-fiber niche, providing new insights into the ecological succession required to restore homeostasis.

## RESULTS

### Fiber fermentation drives robust acidification and SCFA production independent of oxygen stress

**Fig 1:**
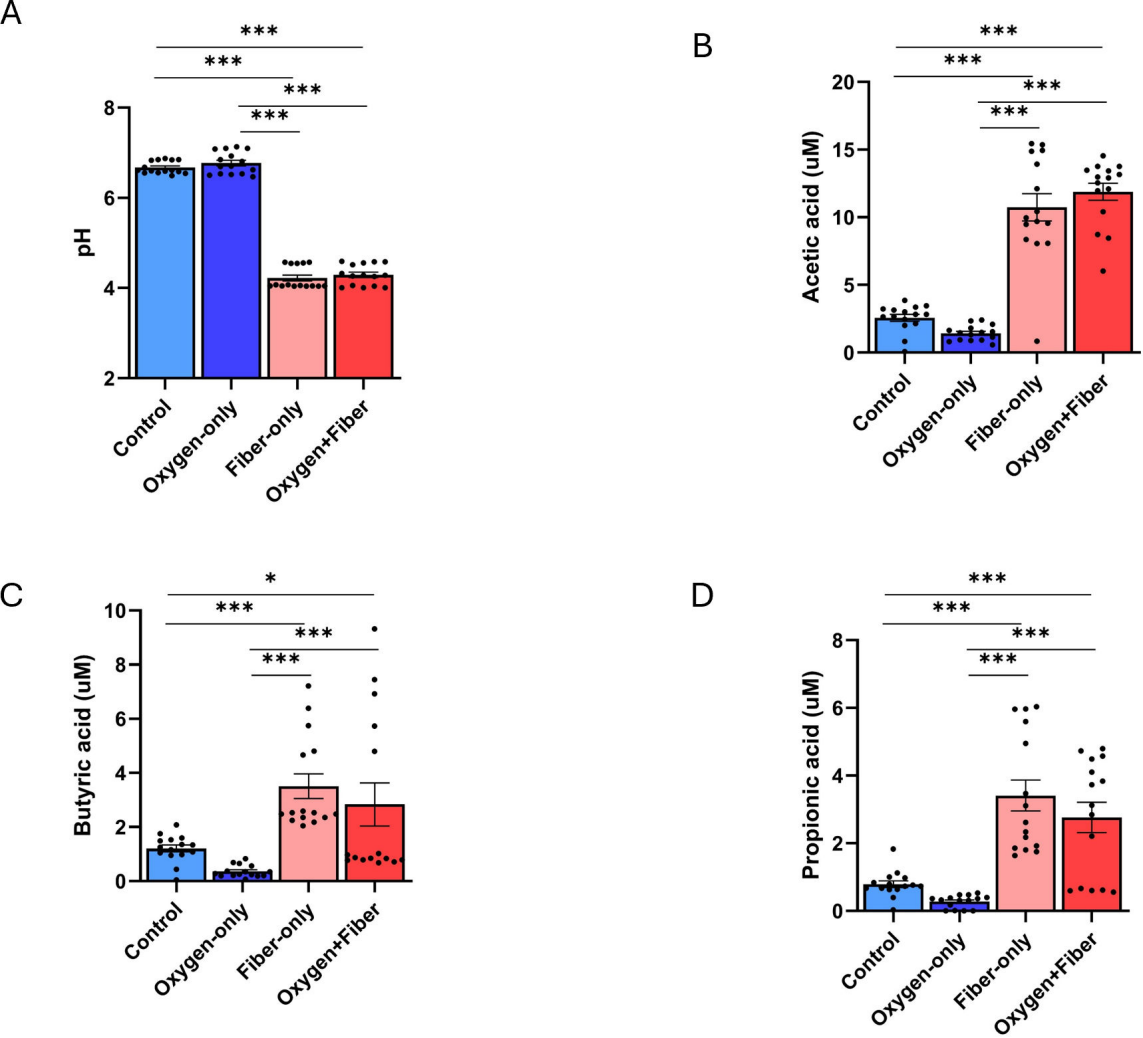
Fiber formula (1% wt/vol) increased major short-chain fatty acid (SCFA) concentrations and decreased pH, even under oxygen exposure. Shown are quantifications of (**A**) pH, (**B**) acetic acid, (**C**) butyric acid, and (**D**) propionic acid. SCFA concentrations were analyzed using a linear mixed-effects model with treatment as a fixed effect and donor as a random effect. Pairwise comparisons were performed using Tukey’s adjustment for multiple testing (***P < 0.001, *P < 0.05).

To determine if dietary fibers could maintain a fermentative environment despite oxygen intrusion, we monitored pH and SCFA production after 24 h of *ex vivo* fermentation. In the control (no oxygen, no fiber) and Oxygen-only groups, the pH remained near neutral (~6.7), indicating a lack of significant fermentative activity (Fig. 1A). However, the addition of the fiber formula (1% wt/vol) triggered a dramatic acidification of the environment, dropping the pH to approximately ~4.2 (Fig. 1A). Crucially, this fiber-driven acidification was resistant to oxygen perturbation; there was no significant difference in pH between the Fiber-only group and the Oxygen+Fiber group.

This acidification was driven by a substantial increase in total SCFA production. Acetic acid, propionic acid, and butyric acid, the three primary microbial fermentation products—were all significantly elevated in fiber-treated groups compared to non-fiber controls. Specifically, acetic acid reached its highest concentrations in the Oxygen+Fiber treatment, while propionic and butyric acids showed similar robust elevations (Fig. 1B through D). Notably, the presence of 5% oxygen did not significantly dampen the production of these major SCFAs when fiber was present. This suggests that the fiber formula supports a metabolic network capable of sustaining fermentation even under microaerophilic stress.

### Dietary fiber supplementation prevents oxygen-driven structural dysbiosis

**Fig 2:**
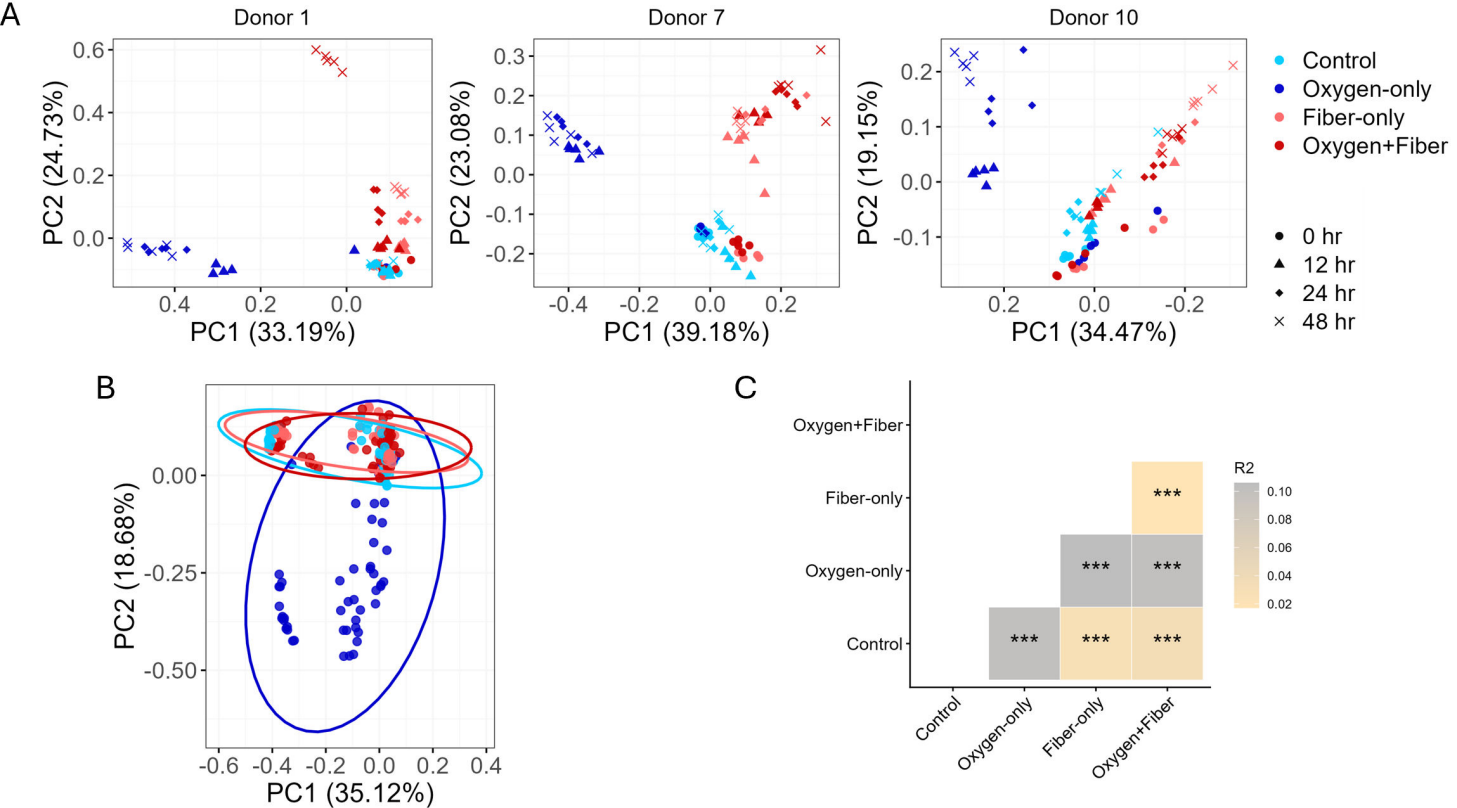
Fiber formula (1% wt/vol) mitigated oxygen-induced disruption of the gut microbiota structure. (**A**) PCoA plot based on Bray-Curtis dissimilarity metric at the ASV-level, visualizing gut microbiota structure for three individual donors. (**B**) Subject-adjusted PCoA plot of the ASVs based on the Bray-Curtis dissimilarity metric, integrating the samples from all the donors. (**C**) Pairwise subject-stratified PERMANOVA (999 permutations); *** indicating Benjamini-Hochberg adjusted *P* < 0.001.

We next evaluated whether the chemical resilience provided by fiber translated to structural resilience of the microbiome. We sequenced the 16S rRNA gene V4 region from 240 samples across four time points (0, 12, 24, and 48 h), identifying 1,516 amplicon sequence variants (ASVs). Principal coordinate analysis (PCoA) based on Bray-Curtis dissimilarity revealed that oxygen exposure acted as a strong selective pressure (Fig. 2A). In all three donors, samples from the Oxygen-only group shifted distinctly away from the Control group along the first principal coordinate (PC1), indicating a major restructuring of the community.

However, the addition of fiber effectively neutralized this shift. The microbial community structure of the Oxygen+Fiber group clustered tightly with the Fiber-only group, maintaining a trajectory distinct from the Oxygen-only phenotype. When we adjusted for inter-individual variation using subject-stratified analysis, the pattern remained consistent: oxygen drove a directional shift (primarily along PC2) indicative of dysbiosis, while fiber supplementation “anchored” the community, preventing the oxygen-induced drift (Fig. 2B and C). This indicates that the provision of fermentable substrates is sufficient to stabilize the global microbiome structure against oxygen stress in a host-independent manner.

### Guild-based analysis captures functional ecological units

**Fig 3:**
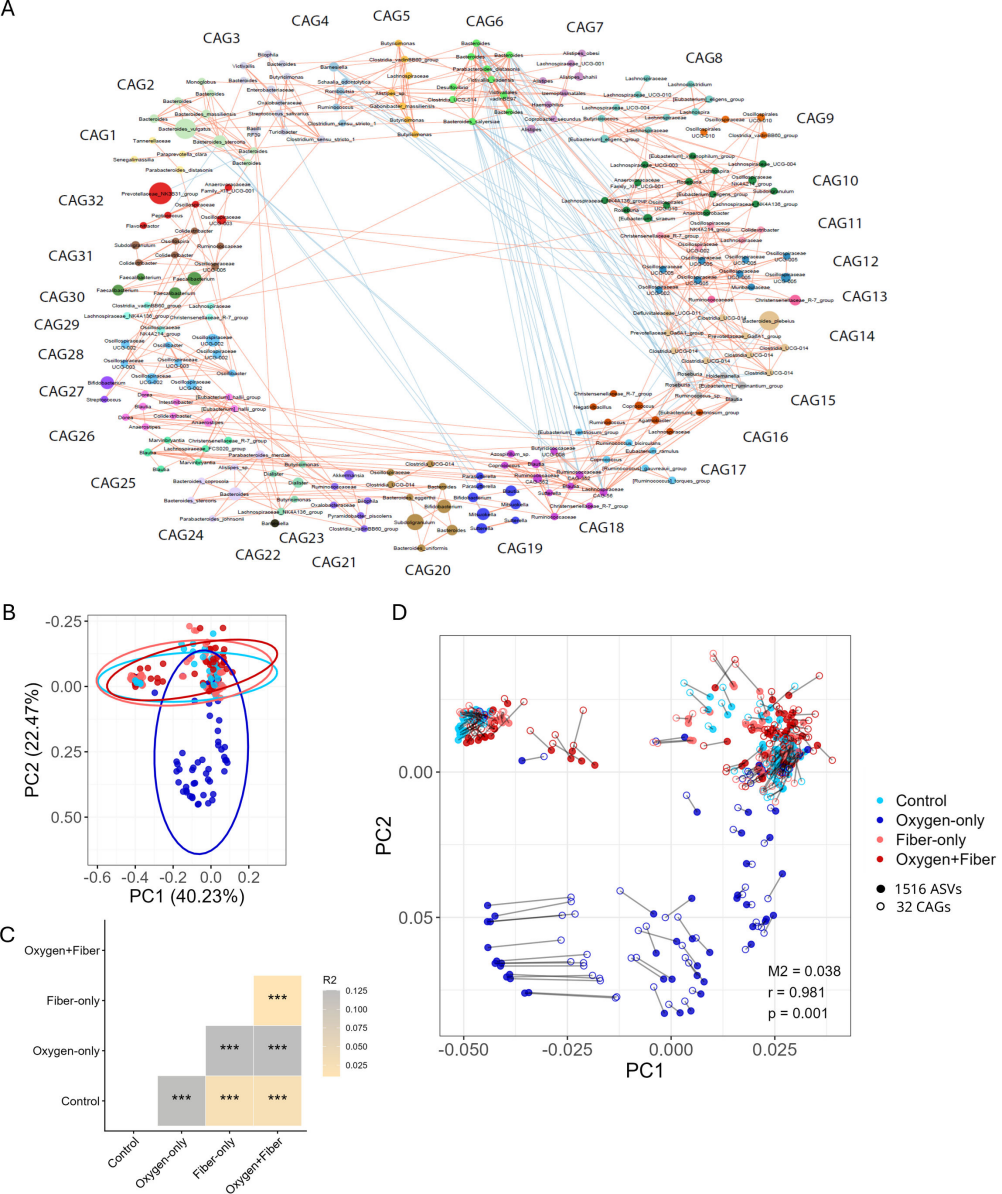
CAGs captured gut microbiota structure and indicated that the fiber formula (1% wt/vol) mitigates oxygen-induced disruption. (**A**) Co-abundance network of prevalent ASVs. Red and blue edges indicate significant positive and negative correlations, respectively. Node size represents the mean ASV abundance. (|r| > 0.5, *P* < 0.05) are shown. (**B**) Subject-adjusted principal coordinate analysis (aPCoA) plot of Bray-Curtis dissimilarity metric integrating the samples from all the donors at the CAG-level. (**C**) Pairwise subject-stratified PERMANOVA (999 permutations); *** indicating Benjamini-Hochberg adjusted *P* < 0.001. (**D**) Procrustes analysis comparing aPCoA ordinations for the 1,516 ASVs and the 32 CAGs based on Bray-Curtis dissimilarity.

To move beyond standard taxonomy and dissect the specific bacterial interactions driving these shifts, we employed a guild-based analytical framework. Using co-abundance criteria, we grouped the 270 most prevalent ASVs (30% prevalence accounting for ~95% of total abundance) into 32 co-abundance groups (CAGs) or “guilds” (Fig. 3A).

To validate that these guilds accurately represented the community dynamics, we performed a covariate-adjusted principal coordinates analysis (aPCoA) using the 32 CAG abundances. The resulting ordination patterns closely mirrored those generated from the ASV-level data. A Procrustes analysis confirmed this high concordance with a significant correlation (*P* = 0.001), demonstrating that the guild-level framework effectively reduced the dimensionality of the data while preserving the underlying ecological signals. Consequently, we utilized these CAGs to identify specific responders to oxygen and fiber treatments.

### Fiber fermentation chemically suppresses oxygen-tolerant pathobiont guilds

**Fig 4:**
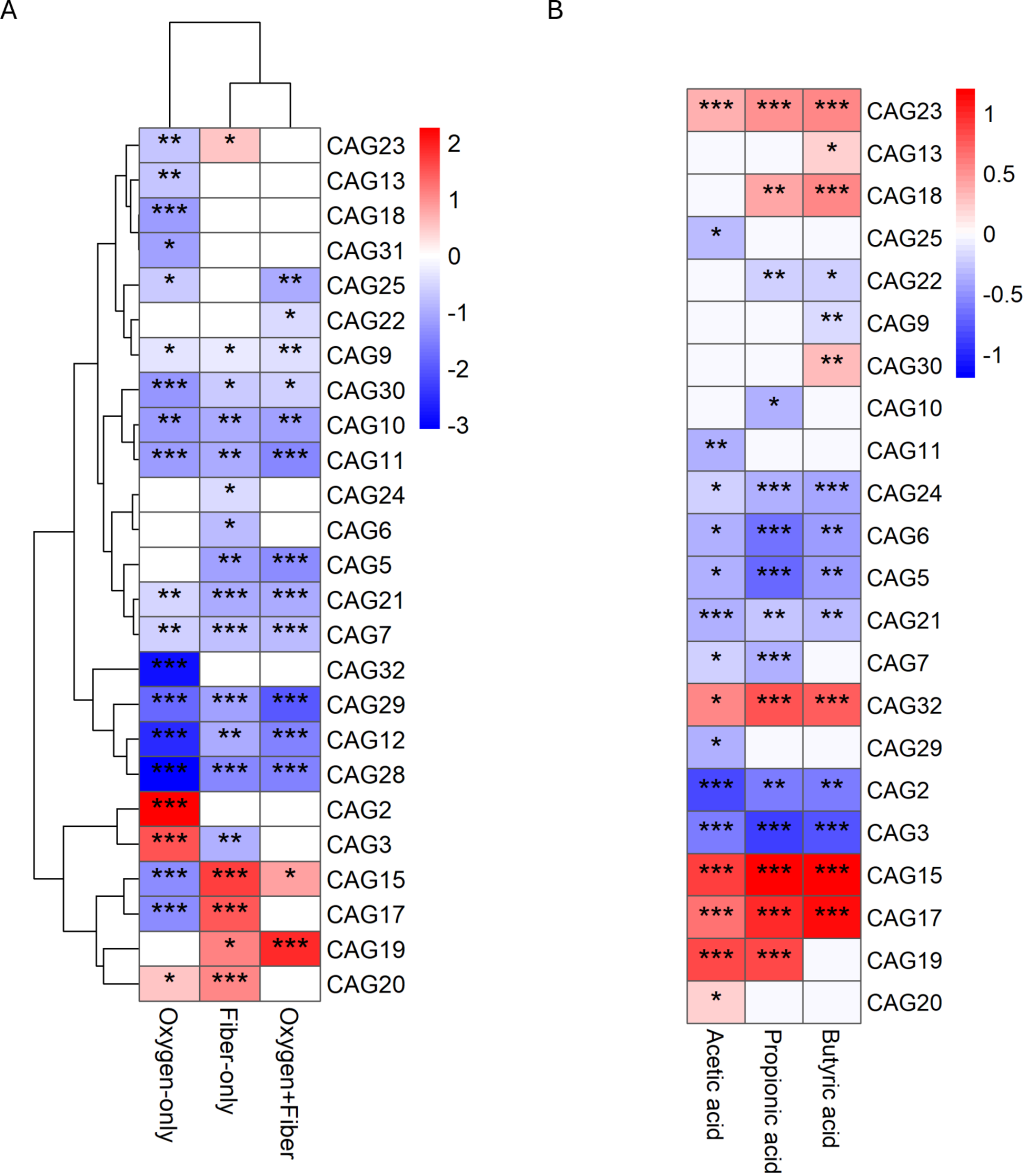
CAGs promoted by oxygen (5% vol/vol) exposure were reduced by fiber formula (1% wt/vol), consistent with SCFA profiles. (**A**) Heatmap of MaAsLin2 regression coefficients showing associations between CAGs and experimental groups relative to the reference group (no oxygen, no fiber). CAG abundances were log-transformed, and the subject was included as a random effect. Red and blue indicate increased or decreased CAG abundance relative to the Control group, respectively. Treatment groups: Oxygen-only, Fiber-only, and Oxygen+Fiber. (**B**) Heatmap shows the standardized coefficients from MaAsLin2 modeling the association between individual CAG abundances and SCFAs. Red indicates a positive association, and blue indicates a negative association. Only CAGs that were significant in panel A are displayed in panel B to facilitate comparison. Significant associations are indicated by asterisks (*q < 0.05, **q < 0.01, ***q < 0.001) corresponding to the FDR-adjusted q-values.

**Fig 5:**
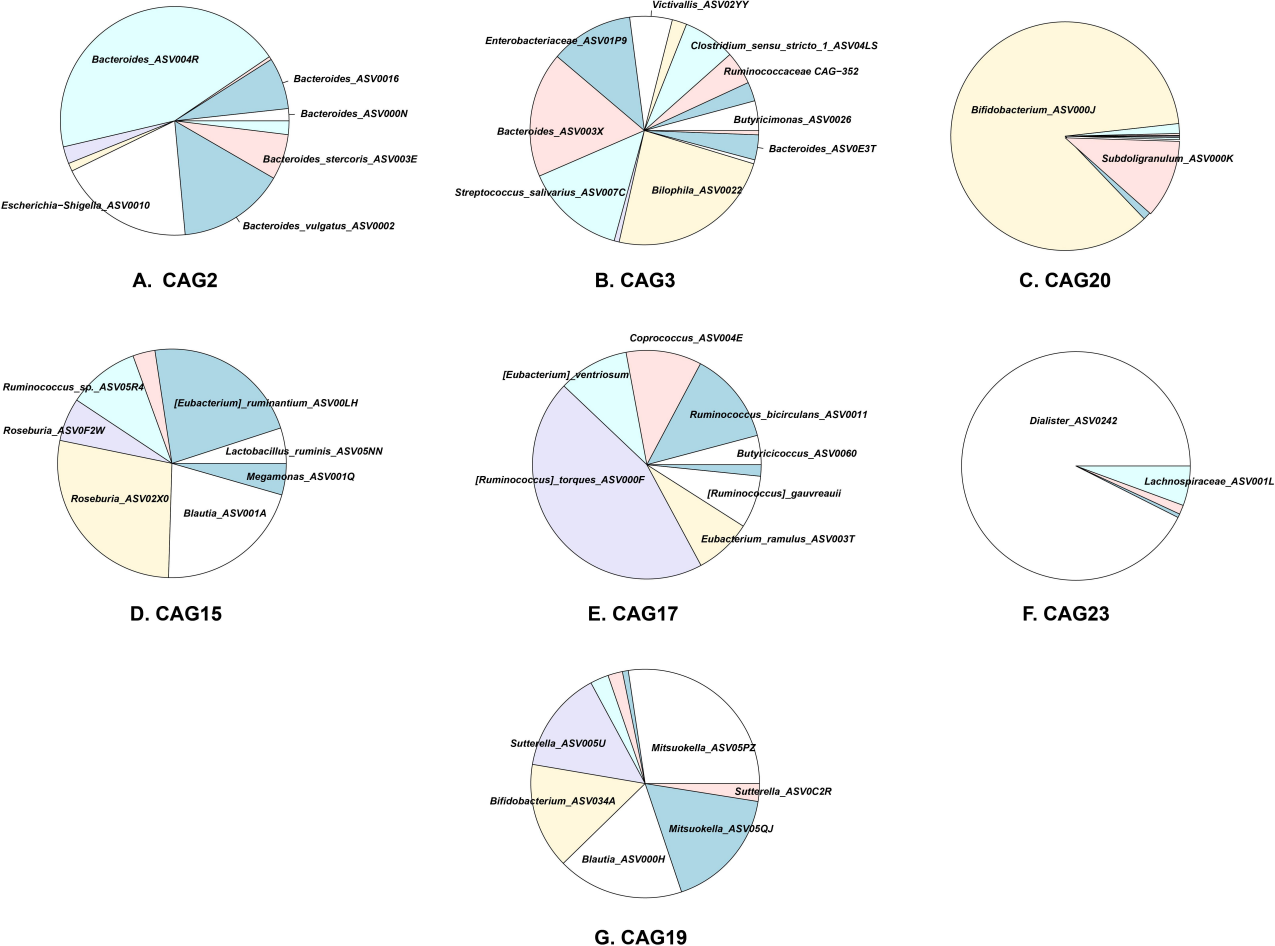
Key CAGs responding to oxygen exposure (5% vol/vol) and fiber formula (1% wt/vol) treatments. Shown is the ASV composition of key CAGs after 24 h of fermentation under each oxygen-fiber treatment condition. ASVs contributing less than 10% to a CAG’s total abundance are not shown.

With MaAsLin2 analysis, we identified two specific guilds, CAG2 and CAG3, that functioned as opportunistic pathobionts (Fig. 4A). In the absence of fiber, these guilds were significantly enriched by oxygen exposure. CAG2 was dominated by *Escherichia-Shigella* (ASV0010) and several *Bacteroides* species (*B. vulgatus* ASV0002, *B. stercoris* ASV003E) (Fig. 5A). In the Oxygen-only group, *Escherichia-Shigella* (ASV0010) bloomed to comprise 5%–50% of the total microbial community across donors. Similarly, CAG3, which contained facultative anaerobes, such as Enterobacteriaceae (ASV01P9) and *Streptococcus* (ASV007C), dominated the Oxygen-only environment (Fig. 5B).

However, the addition of the fiber formula effectively suppressed these blooms. In the Oxygen+Fiber group, the relative abundance of *Escherichia-Shigella* (ASV0010) was reduced to *≤*0.1%, and CAG3 was similarly suppressed. MaAsLin2 analysis confirmed a significant negative association between these guilds and the fiber treatment. Furthermore, both CAG2 and CAG3 showed strong negative correlations with acetic, propionic, and butyric acids (Fig. 4B). This supports the hypothesis that fiber-driven acidification and SCFA accumulation create a chemical environment that excludes these pH-sensitive facultative anaerobes, even when oxygen is available.

### Fiber rescues oxygen-sensitive guilds

Conversely, we identified a set of guilds comprised of obligate anaerobes that were highly sensitive to oxygen but responsive to fiber. CAG15, CAG17, and CAG23 were significantly depleted in the Oxygen-only group, confirming their vulnerability to oxidative stress (Fig. 4A). Taxonomically, these guilds contained key butyrate producers: CAG15 included *Roseburia* (ASV02X0), *Eubacterium* (ASV00LH), and *Blautia* (ASV001A); CAG17 was composed of *Ruminococcus* (ASV000F); and CAG23 was dominated by *Dialister* (ASV0242) (Fig. 5D and E).

Strikingly, the addition of fiber rescued these guilds from oxygen-induced depletion (Fig. 4A). In the Oxygen+Fiber group, the abundances of CAG15, CAG17, and CAG23 were restored to levels comparable to or exceeding the control (Fig. 4A). This suggests that the metabolic benefits of fiber fermentation, likely through the generation of a reducing environment or cross-feeding—outweighed the inhibitory effects of oxygen. Consistent with their roles as primary fermenters, all three guilds showed significant positive correlations with major SCFAs.

### A distinct “transitional guild” (CAG19) thrives specifically in the oxygen-fiber niche

Beyond the simple dichotomy of “suppressed pathobionts” and “rescued anaerobes,” we discovered a unique guild, CAG19, with a distinct ecological signature. It was most strongly promoted only when both oxygen and fiber were present (the Oxygen+Fiber group) (Fig. 4A).

CAG19 was positively correlated with acetate and propionate production. Compositionally, it contained *Bifidobacterium* (ASV034A), *Blautia* (ASV000H), and *Mitsuokella* (ASV05PZ) (Fig. 5G). This distinct response pattern contrasts with another *Bifidobacterium*-containing guild, CAG20 (dominated by ASV000J), which responded positively to fiber but lost its significance in the combined Oxygen+Fiber group (Fig. 4A and 5C). The unique expansion of CAG19 suggests it may function as a “transitional guild” aerotolerant fermenters that can utilize complex carbohydrates while enduring (or potentially scavenging) oxygen, thereby helping to restore the anaerobic niche for the foundation guilds.

## DISCUSSION

In this study, we provide direct *ex vivo* evidence that the gut microbiota possesses an intrinsic capacity to manage oxygen stress when fueled by dietary fibers. While the “Oxygen Hypothesis” typically posits that dysbiosis is driven by the loss of host-mediated epithelial hypoxia, our findings demonstrate that dietary fibers can reverse this process independent of host cells. We showed that (i) oxygen alone induces a dysbiotic shift favoring facultative anaerobes; (ii) fiber fermentation generates a chemical environment (high SCFA, low pH) that suppresses these pathobionts even in the presence of oxygen; and (iii) this environment effectively rescues key obligate anaerobes. This suggests that fiber acts as a “metabolic shield,” allowing the microbiome to autoregulate its niche against oxidative stress.

A critical finding was the suppression of CAG2 (*Escherichia-Shigella*) and CAG3 (Enterobacteriaceae) in the Oxygen+Fiber treatment. These facultative anaerobes are classic markers of dysbiosis in IBD and metabolic syndrome (27, 28). While oxygen typically fuels their expansion, our data confirms that their bloom is conditional on a non-acidic environment. As noted by Sorbara et al., SCFAs—particularly acetate—are toxic to Enterobacteriaceae at low pH due to intracellular acidification (29). In our study, the fiber formula drove pH down to ~4.2 while boosting acetate production. This confirms that the fiber-mediated restoration of colonization resistance is primarily chemical: the acidic fermentative output creates a hostile niche that overrides the growth advantage oxygen would otherwise provide to these pathobionts.

The rescue of the anaerobic guilds—CAG15 (*Roseburia, Blautia*) and CAG17 (*Ruminococcus*)—in the Oxygen+Fiber group is particularly significant. These obligate anaerobes are typically highly sensitive to oxidative stress. Their survival suggests that the “transitional guilds” (like CAG19) and the chemical changes induced by fiber fermentation effectively detoxify the microenvironment. Additionally, these taxa may possess specific stress-response mechanisms (30, 31), such as the activation of PerR regulons or sigma factors (32), which allow them to endure microaerophilic conditions long enough to re-establish an anaerobic niche. This implies that “strict” anaerobes are more resilient than previously thought, provided they are supported by a robust guild network and adequate substrate availability.

Our results underscore the necessity of moving beyond taxonomy to a guild-based framework. Taxon-based analyses often yield conflicting results; for example, *B. vulgatus* has been linked to both health and disease (33, 34), and *E. coli* associations with UC are inconsistent (35). These contradictions likely stem from strain-level functional diversity. This was illustrated by our identification of CAG19. While standard taxonomy would simply report an overall increase in *Bifidobacterium*, our analysis distinguished between the *Bifidobacterium* in CAG20 (oxygen-tolerant) and the oxygen-sensitive *Bifidobacterium* (ASV034A) in CAG19, which thrived specifically in the Oxygen+Fiber niche. CAG19 appears to function as a “transitional guild”—aerotolerant fermenters (36) that utilize fiber to produce acetate and lactate (37). This early fermentative activity likely shields oxygen stress and lowers pH, paving the way for the strict anaerobes. By clustering bacteria based on co-abundance rather than phylogeny, guild-based analysis captures these coherent functional units that drive ecological succession.

Host-dependent oxygen regulation has been well characterized *in vivo*, butyrate oxidation by colonocytes drives epithelial hypoxia, thereby suppressing the expansion of facultative pathobionts under physiological conditions (13, 17). Chemical oxygen consumption pathways along the gut lumen have also been mapped using integrated modeling and *in vivo* measurements, highlighting how host-derived oxygen shapes microbial biogeography. More recently, an *ex vivo* study demonstrated that elemental iron could protect human fecal microbiota from oxygen-induced dysbiosis by chemically scavenging oxygen and reactive oxygen species (38). These studies collectively define important host-driven and chemical routes for limiting oxygen intrusion. However, it remains unknown whether the gut microbiota itself—given an appropriate nutritional substrate—can autonomously resist oxygen stress in the absence of host tissues. No prior work has directly tested whether fermentable dietary fiber fuels a microbial self-protection mechanism against oxygen exposure, nor has it resolved this protection at a guild level. In particular, the emergence of a *Bifidobacterium*-centered transitional guild uniquely under combined fiber and oxygen conditions, and its potential ecological role in supporting the recovery of strictly anaerobic SCFA-producing guilds, has not been previously described.

There are limitations to the current study. Although our approach accounted for inter-individual variation, the limited number of donors restricted the broader applicability of the findings. Future studies involving larger and more diverse donor populations will be important to validate these observations. In addition, the proposed “transitional” function of CAG19 remains speculative. While our data-driven approach suggests that this guild may act as aerotolerant fermenters bridging oxygen-exposed and anaerobic niches, their precise ecological and metabolic roles remain to be confirmed. Future studies employing metagenomics, metatranscriptomics, targeted metabolomics, and co-culture experiments will be essential to validate these predictions and clarify the mechanistic contributions of CAG19 to community resilience under oxygen and fiber perturbations. Finally, although co-abundance-based CAG analysis represents a key strength of this study, it relies on correlation-based relationships among ASVs across experimental perturbations and may not capture direct interactions or causal mechanisms between taxa, underscoring the need for experimental validation of the functional cohesion inferred within these guilds.

In summary, this study validates a host-independent mechanism for the protective effect of dietary fibers against oxygen stress. By fueling a “transitional guild” that acidifies the gut and shields oxygen, fibers restore the dominance of SCFA-producing obligate anaerobes and chemically exclude oxygen-promoted facultative pathobionts. These findings highlight the power of guild-based analysis in dissecting complex microbial interactions and support the use of targeted high-fiber interventions (the WTP diet and NBT-NM108) (21–23) to manage oxygen-driven dysbiosis in clinical settings.

## MATERIALS AND METHODS

### Dietary fiber preparation

A dietary fiber formula was prepared using oat bran, wheat bran, corn bran, sorghum bran, inulin, and Fibersol-2. Oat bran was purchased from Grain Millers (St. Ansgar, IA, USA), wheat bran was kindly provided by Kansas State University, sorghum bran from Nu Life Market (Scott City, KS, USA), corn bran from Honeyville (Ogden, UT, USA), inulin from Cargill (Wayzata, MN, USA), and Fibersol-2 from ADM (Chicago, IL, USA). Bran components (sorghum, corn, oat, wheat) were mixed in a 1.9:1.3:5.1:1.5 ratio and roasted at 135°C for 5 min. The roasted fiber mix (25 g) was subjected to *in vitro* digestion following a slightly modified protocol of Mishra and Monro (39). Briefly, the fiber mix was boiled in 300 mL of distilled water for 20 min, cooled to room temperature, and treated with 10 mL of pepsin (10 mg/mL in 50 mM HCl, P-7000, Sigma-Aldrich, St Louis, MO, USA) at pH 2.5 for 30 min at 37°C to remove proteins. Subsequently, 8 mL pancreatin (250 mg/mL, P-7545, Sigma, St Louis, MO, USA) and 2 mL of amyloglucosidase (E-AMGDF, Megazyme, Lansing, MI, USA) were added at pH 6.9 and incubated for 6 h at 37°C with shaking at 150 rpm to digest remaining proteins, lipids, and starch. The digested mixture was transferred into dialysis tubing (3.5 kD, Spectrum, Waltham, MA, USA) and dialyzed against distilled water for 72 h. Retentates were frozen at −20°C overnight and then freeze-dried for 72 h. The dried fiber mix was combined with inulin and Fibersol-2 in a 4:1:1 ratio to produce the final dietary fiber formula.

### *Ex vivo* fermentation

Three healthy adults (female, age 57; male, age 65; female, age 22) with no gastrointestinal disorders and no antibiotic use in the preceding 3 months were recruited under Rutgers University IRB protocol #2019000885. Fresh fecal samples were collected using a standardized collection kit and transferred to an anaerobic chamber (5% H₂, 5% CO₂, 90% N₂; Airgas, MO, USA) within 2 h of collection. All donors were self-reported as healthy, reported no regular medication use, and consumed an omnivorous diet; however, detailed dietary intake data were not collected. Donor body mass index values ranged from 18.5 to 24.9. Approximately 50 g of stool from each donor was homogenized with 150 mL phosphate-buffered saline (PBS; pH 7.4; 0.01 M phosphate, 0.0027 M KCl, 0.137 M NaCl; Sigma-Aldrich, St. Louis, MO, USA) containing 0.05% L-cysteine hydrochloride and 1 mg/mL resazurin using a blender. The slurry was filtered through four layers of cheesecloth to remove particulate matter and diluted with four volumes of reduced PBS to generate a 5% (wt/vol) fecal inoculum. For the Fiber-only and Oxygen+Fiber groups, the fecal inoculum was supplemented with 1% (wt/vol) of the fiber formula. Estimates of ~30–40 g/day of microbiota-available carbohydrates (40) and a colonic volume of ~0.4 L (41) correspond to ~75–100 g/L of fermentable substrate *in vivo*. The estimated ~75–100 g/L of fermentable substrate *in vivo* corresponds to ~10% (wt/vol), making our 1% (wt/vol) concentration modest, as these estimates represent maximal luminal concentrations. However, actual substrate availability *in vivo* is heterogeneous, is continuously depleted by microbial fermentation, and is further influenced by gut transit time, all of which reduce the instantaneous pool of digested fiber accessible to microbes. In static batch fermentation systems, 1% (wt/vol) has been widely used (42) to provide sufficient fermentable substrate for microbial metabolism, making it a conservative yet physiologically relevant approximation for *ex vivo* studies. Aliquots were dispensed into Hungate tubes and sealed with butyl rubber stoppers inside the anaerobic chamber. Five biological replicates were prepared per treatment at each time point. For the Oxygen-only and Oxygen+Fiber groups, sterile air was injected into sealed tubes to achieve a final concentration of 5% O₂ (vol/vol). All cultures were incubated at 37°C with shaking at 100 rpm. Samples were collected at 0, 12, 24, and 48 h, centrifuged, and separated into supernatant and pellet fractions. Supernatants were used for pH and SCFA measurements, and pellets were processed for 16S rRNA gene V4 region sequencing.

### SCFA analysis

SCFAs were extracted and quantified as described previously (43). Briefly, samples were homogenized for 1 min at 22.5 rpm in 30 mM hydrochloric acid containing 10 mM methyl-heptanoate as an internal standard. Water was added to a final volume of 400 µL, after which 250 µL methyl tert-butyl ether was added. Samples were vortexed, incubated at 4°C for 5 min, and vortexed again. Phase separation was achieved by centrifugation at 500 × *g* for 3 min at room temperature. The upper organic phase was transferred to autosampler vials for GC–MS. SCFAs were analyzed on an Agilent 7890B gas chromatograph coupled to a 5977B mass spectrometer equipped with a DB-WAX column (30 m × 0.25 mm × 0.25 µm; Agilent 122-7032) using helium as the carrier gas. Chromatograms were processed with MassHunter Quantitative Analysis (version B.07.06.2704), and SCFA concentrations are reported in µM (Table S1).

### DNA extraction and sequencing

Fecal pellets were extracted using a modified Q protocol (44), substituting the TissueLyser II (Qiagen) at 25 Hz for mechanical lysis and eluting DNA in nuclease-free water instead of AE buffer. The 16S rRNA gene’s V4 region was amplified with 515F and 806R (45, 46) and was sequenced using the Ion GeneStudio S5 (ThermoFisher Scientific) following the manufacturer’s instructions, as described previously (23).

### Microbiota data processing and statistical analysis

A total of 240 fermented samples (4 treatment groups × 4 time points × 5 replicates × 3 donors) were collected for 16S rRNA gene sequencing targeting the V4 region, yielding ~3 million high-quality reads. Primer sequences were trimmed using cutadapt (47) within QIIME2 software (48). Denoising was performed with the dada2 denoise-single workflow using parameters –p-trim-left 0 and –p-trunc-len 215, generating ASVs (49) that were obtained, and spurious ASVs were removed by abundance filtering (50). Taxonomic assignment was conducted with the QIIME2 q2-feature-classifier plugin (51) based on the SILVA database (release 132) (51). Sequencing data were rarefied to 28,000 reads per sample based on the rarefaction curve assessment. Pairwise correlations among ASVs with >30% prevalence were calculated using the Bland and Altman repeated-measurement procedure (52). Correlation matrices were transformed into distance matrices (1—correlation) and clustered using Ward’s hierarchical algorithm to generate a dendrogram. PERMANOVA (9,999 permutations; *P* < 0.001) was then applied sequentially from the top of the tree to define significant clades (CAGs). CAG abundances were calculated by summing the ASVs within each clade, and the resulting CAG network was visualized in Cytoscape (v3.9.0). Statistical analysis was performed in R version 4.5.1. Covariate-aPCoA was performed on Bray-Curtis distances of CAGs and ASVs, adjusting for donor effects, to visualize treatment effects along the first two principal coordinates using the R packages *vegan* and *aPCoA* (53). Procrustes and PROTEST analyses were conducted with the R package *vegan*. MaAsLin2 was used to assess correlations and significance between CAG abundances and treatments (with the control as reference), as well as between CAG abundances and SCFA concentrations (54). Figures were generated with R packages *ggplot2* and *pheatmap*. SCFA and pH data were visualized in GraphPad Prism 9 (San Diego, CA, USA). SCFA concentrations and pH were compared between groups using a linear mixed-effects model with treatment as a fixed effect and donor as a random effect. Pairwise comparisons were adjusted for multiple testing using Tukey’s method with the *lme4* package in R.

## Data Availability

The 16S rRNA gene V4 region sequence data generated in this study have been submitted to Sequence Read Archive (SRA) maintained by NCBI under the accession number PRJNA1390810.

## SUPPLEMENTAL MATERIAL

Table S1 mbio.03934-25-s0001.xlsx SCFA concentrations.

ASM does not own the copyrights to Supplemental Material that may be linked to, or accessed through, an article. The authors have granted ASM a non-exclusive, world-wide license to publish the Supplemental Material files. Please contact the corresponding author directly for reuse.

## References

[1] Ni J, Wu GD, Albenberg L, Tomov VT. 2017. Gut microbiota and IBD: causation or correlation? Nat Rev Gastroenterol Hepatol 14:573–584. doi:10.1038/nrgastro.2017.88 28743984 PMC5880536

[2] Morgan XC, Tickle TL, Sokol H, Gevers D, Devaney KL, Ward DV, Reyes JA, Shah SA, LeLeiko N, Snapper SB, Bousvaros A, Korzenik J, Sands BE, Xavier RJ, Huttenhower C. 2012. Dysfunction of the intestinal microbiome in inflammatory bowel disease and treatment. Genome Biol 13:1–18. doi:10.1186/gb-2012-13-9-r79 PMC3506950 23013615

[3] Manichanh C, Borruel N, Casellas F, Guarner F. 2012. The gut microbiota in IBD. Nat Rev Gastroenterol Hepatol 9:599–608. doi:10.1038/nrgastro.2012.152 22907164

[4] Guss JD, Ziemian SN, Luna M, Sandoval TN, Holyoak DT, Guisado GG, Roubert S, Callahan RL, Brito IL, van der Meulen MCH, Goldring SR, Hernandez CJ. 2019. The effects of metabolic syndrome, obesity, and the gut microbiome on load-induced osteoarthritis. Osteoarthr Cartil 27:129–139. doi:10.1016/j.joca.2018.07.020 PMC6309743 30240938

[5] Camilleri M. 2019. Leaky gut: mechanisms, measurement and clinical implications in humans. Gut 68:1516–1526. doi:10.1136/gutjnl-2019-318427 31076401 PMC6790068

[6] Kaplan GG, Ng SC. 2017. Understanding and preventing the global increase of inflammatory bowel disease. Gastroenterology 152:313–321. doi:10.1053/j.gastro.2016.10.020 27793607

[7] Miquel S, Leclerc M, Martin R, Chain F, Lenoir M, Raguideau S, Hudault S, Bridonneau C, Northen T, Bowen B, Bermúdez-Humarán LG, Sokol H, Thomas M, Langella P. 2015. Identification of metabolic signatures linked to anti-inflammatory effects of Faecalibacterium prausnitzii. mBio 6:e00300-15. doi:10.1128/mBio.00300-15 25900655 PMC4453580

[8] M’Koma AE. 2013. Inflammatory bowel disease: an expanding global health problem. Clin Med Insights Gastroenterol 6:33–47. doi:10.4137/CGast.S12731 24833941 PMC4020403

[9] Gupta N, Shah P, Nayyar S, Misra A. 2013. Childhood obesity and the metabolic syndrome in developing countries. Indian J Pediatr 80 Suppl 1:S28–S37. doi:10.1007/s12098-012-0923-5 23334584

[10] Ananthakrishnan AN. 2015. Epidemiology and risk factors for IBD. Nat Rev Gastroenterol Hepatol 12:205–217. doi:10.1038/nrgastro.2015.34 25732745

[11] Di Marzo V, Silvestri C. 2019. Lifestyle and metabolic syndrome: contribution of the endocannabinoidome. Nutrients 11:1956. doi:10.3390/nu11081956 31434293 PMC6722643

[12] Anderson JW, Baird P, Davis RH Jr, Ferreri S, Knudtson M, Koraym A, Waters V, Williams CL. 2009. Health benefits of dietary fiber. Nutr Rev 67:188–205. doi:10.1111/j.1753-4887.2009.00189.x 19335713

[13] Rivera-Chávez F, Lopez CA, Bäumler AJ. 2017. Oxygen as a driver of gut dysbiosis. Free Radic Biol Med 105:93–101. doi:10.1016/j.freeradbiomed.2016.09.022 27677568

[14] Litvak Y, Byndloss MX, Bäumler AJ. 2018. Colonocyte metabolism shapes the gut microbiota. Science 362:eaat9076. doi:10.1126/science.aat9076 30498100 PMC6296223

[15] Litvak Y, Byndloss MX, Tsolis RM, Bäumler AJ. 2017. Dysbiotic Proteobacteria expansion: a microbial signature of epithelial dysfunction. Curr Opin Microbiol 39:1–6. doi:10.1016/j.mib.2017.07.003 28783509

[16] Rigottier-Gois L. 2013. Dysbiosis in inflammatory bowel diseases: the oxygen hypothesis. ISME J 7:1256–1261. doi:10.1038/ismej.2013.80 23677008 PMC3695303

[17] Wu H, Zeng W, Dai N, Gu J, He Y, Qin H, Lin L, Fu X, Fu B, Xing Z. 2025. Hyperoxia as a driver of gut dysbiosis. Front Microbiol 16:1675652. doi:10.3389/fmicb.2025.1675652 41321832 PMC12657494

[18] Videla S, Vilaseca J, Antolín M, García-Lafuente A, Guarner F, Crespo E, Casalots J, Salas A, Malagelada JR. 2001. Dietary inulin improves distal colitis induced by dextran sodium sulfate in the rat. Am J Gastroenterol 96:1486–1493. doi:10.1111/j.1572-0241.2001.03802.x 11374687

[19] Rodríguez-Cabezas ME, Gálvez J, Lorente MD, Concha A, Camuesco D, Azzouz S, Osuna A, Redondo L, Zarzuelo A. 2002. Dietary fiber down-regulates colonic tumor necrosis factor α and nitric oxide production in trinitrobenzenesulfonic acid-induced colitic rats. J Nutr 132:3263–3271. doi:10.1093/jn/132.11.3263 12421838

[20] Kanauchi O, Serizawa I, Araki Y, Suzuki A, Andoh A, Fujiyama Y, Mitsuyama K, Takaki K, Toyonaga A, Sata M, Bamba T. 2003. Germinated barley foodstuff, a prebiotic product, ameliorates inflammation of colitis through modulation of the enteric environment. J Gastroenterol 38:134–141. doi:10.1007/s005350300022 12640526

[21] Zhang C, Yin A, Li H, Wang R, Wu G, Shen J, Zhang M, Wang L, Hou Y, Ouyang H, et al.. 2015. Dietary modulation of gut microbiota contributes to alleviation of both genetic and simple obesity in children. EBioMedicine 2:968–984. doi:10.1016/j.ebiom.2015.07.007 26425705 PMC4563136

[22] Zhao L, Zhang F, Ding X, Wu G, Lam YY, Wang X, Fu H, Xue X, Lu C, Ma J, et al.. 2018. Gut bacteria selectively promoted by dietary fibers alleviate type 2 diabetes. Science 359:1151–1156. doi:10.1126/science.aao5774 29590046

[23] Wang Y, Wu G, Zhao L, Wang W. 2022. Nutritional modulation of gut microbiota alleviates severe gastrointestinal symptoms in a patient with post-acute COVID-19 syndrome. mBio 13:e0380121. doi:10.1128/mbio.03801-21 35254129 PMC9040862

[24] Zhang C, Zhao L. 2016. Strain-level dissection of the contribution of the gut microbiome to human metabolic disease. Genome Med 8:41. doi:10.1186/s13073-016-0304-1 27098841 PMC4839137

[25] Wu G, Zhao N, Zhang C, Lam YY, Zhao L. 2021. Guild-based analysis for understanding gut microbiome in human health and diseases. Genome Med 13:22. doi:10.1186/s13073-021-00840-y 33563315 PMC7874449

[26] Espey MG. 2013. Role of oxygen gradients in shaping redox relationships between the human intestine and its microbiota. Free Radic Biol Med 55:130–140. doi:10.1016/j.freeradbiomed.2012.10.554 23127782

[27] Dubourg G, Lagier J-C, Hüe S, Surenaud M, Bachar D, Robert C, Michelle C, Ravaux I, Mokhtari S, Million M, Stein A, Brouqui P, Levy Y, Raoult D. 2016. Gut microbiota associated with HIV infection is significantly enriched in bacteria tolerant to oxygen. BMJ Open Gastroenterol 3:e000080. doi:10.1136/bmjgast-2016-000080 PMC4985784 27547442

[28] Carroll IM, Ringel‐Kulka T, Siddle JP, Ringel Y. 2012. Alterations in composition and diversity of the intestinal microbiota in patients with diarrhea‐predominant irritable bowel syndrome. Neurogastroenterology Motil 24:521. doi:10.1111/j.1365-2982.2012.01891.x PMC3975596 22339879

[29] Sorbara MT, Dubin K, Littmann ER, Moody TU, Fontana E, Seok R, Leiner IM, Taur Y, Peled JU, van den Brink MRM, Litvak Y, Bäumler AJ, Chaubard J-L, Pickard AJ, Cross JR, Pamer EG. 2019. Inhibiting antibiotic-resistant Enterobacteriaceae by microbiota-mediated intracellular acidification. J Exp Med 216:84–98. doi:10.1084/jem.20181639 30563917 PMC6314524

[30] Liu X, Mao B, Gu J, Wu J, Cui S, Wang G, Zhao J, Zhang H, Chen W. 2021. Blautia-a new functional genus with potential probiotic properties? Gut Microbes 13:1–21. doi:10.1080/19490976.2021.1875796 PMC7872077 33525961

[31] Crost EH, Coletto E, Bell A, Juge N. 2023. Ruminococcus gnavus: friend or foe for human health. FEMS Microbiol Rev 47:fuad014. doi:10.1093/femsre/fuad014 37015876 PMC10112845

[32] Morvan C, Folgosa F, Kint N, Teixeira M, Martin-Verstraete I. 2021. Responses of Clostridia to oxygen: from detoxification to adaptive strategies. Environ Microbiol 23:4112–4125. doi:10.1111/1462-2920.15665 34245087

[33] Xu M, Lan R, Qiao L, Lin X, Hu D, Zhang S, Yang J, Zhou J, Ren Z, Li X, Liu G, Liu L, Xu J. 2023. Bacteroides vulgatus ameliorates lipid metabolic disorders and modulates gut microbial composition in hyperlipidemic rats. Microbiol Spectr 11:e0251722. doi:10.1128/spectrum.02517-22 36625637 PMC9927244

[34] Mills RH, Dulai PS, Vázquez-Baeza Y, Sauceda C, Daniel N, Gerner RR, Batachari LE, Malfavon M, Zhu Q, Weldon K, Humphrey G, Carrillo-Terrazas M, Goldasich LD, Bryant M, Raffatellu M, Quinn RA, Gewirtz AT, Chassaing B, Chu H, Sandborn WJ, Dorrestein PC, Knight R, Gonzalez DJ. 2022. Multi-omics analyses of the ulcerative colitis gut microbiome link Bacteroides vulgatus proteases with disease severity. Nat Microbiol 7:262–276. doi:10.1038/s41564-021-01050-3 35087228 PMC8852248

[35] Martinez-Medina M, Garcia-Gil LJ. 2014. Escherichia coli in chronic inflammatory bowel diseases: an update on adherent invasive Escherichia coli pathogenicity. World J Gastrointest Pathophysiol 5:213–227. doi:10.4291/wjgp.v5.i3.213 25133024 PMC4133521

[36] Zhang W, Wang Y, Li K, Kwok L-Y, Liu W, Zhang H. 2020. Short communication: Modulation of fatty acid metabolism improves oxygen tolerance of Bifidobacterium animalis ssp. lactis Probio-M8. J Dairy Sci 103:8791–8795. doi:10.3168/jds.2019-18049 32861486

[37] Oude Elferink SJ, Krooneman J, Gottschal JC, Spoelstra SF, Faber F, Driehuis F. 2001. Anaerobic conversion of lactic acid to acetic acid and 1, 2-propanediol by Lactobacillus buchneri. Appl Environ Microbiol 67:125–132. doi:10.1128/AEM.67.1.125-132.2001 11133436 PMC92530

[38] Ostrov I, Gong Y, Zuk JB, Wickramasinghe PCK, Tmenova I, Roopchand DE, Zhao L, Raskin I. 2024. Elemental iron protects gut microbiota against oxygen-induced dysbiosis. PLoS One 19:e0298592. doi:10.1371/journal.pone.0298592 38412144 PMC10898728

[39] Mishra S, Monro JA. 2009. Digestibility of starch fractions in wholegrain rolled oats. J Cereal Sci 50:61–66. doi:10.1016/j.jcs.2009.03.002

[40] Arnoldini M, Sharma R, Moresi C, Chure G, Chabbey J, Slack E, Cremer J. 2025. Quantifying the varying harvest of fermentation products from the human gut microbiota. Cell 188:5332–5342. doi:10.1016/j.cell.2025.07.005 40744013 PMC12556654

[41] Sender R, Fuchs S, Milo R. 2016. Revised estimates for the number of human and bacteria cells in the body. PLoS Biol 14:e1002533. doi:10.1371/journal.pbio.1002533 27541692 PMC4991899

[42] Khan KM, Edwards CA. 2002. Effect of substrate concentration on short chain fatty acid production in in vitro cultures of human faeces with lactulose, a rapidly fermented carbohydrate. Microb Ecol Health Dis 14:160–164. doi:10.1080/089106002320644348

[43] den Hartigh LJ, Gao Z, Goodspeed L, Wang S, Das AK, Burant CF, Chait A, Blaser MJ. 2018. Obese mice losing weight due to trans-10, cis-12 conjugated linoleic acid supplementation or food restriction harbor distinct gut microbiota. J Nutr 148:562–572. doi:10.1093/jn/nxy011 29659960 PMC6251681

[44] Costea PI, Zeller G, Sunagawa S, Pelletier E, Alberti A, Levenez F, Tramontano M, Driessen M, Hercog R, Jung F-E, et al.. 2017. Towards standards for human fecal sample processing in metagenomic studies. Nat Biotechnol 35:1069–1076. doi:10.1038/nbt.3960 28967887

[45] Parada AE, Needham DM, Fuhrman JA. 2016. Every base matters: assessing small subunit rRNA primers for marine microbiomes with mock communities, time series and global field samples. Environ Microbiol 18:1403–1414. doi:10.1111/1462-2920.13023 26271760

[46] Apprill A, McNally S, Parsons R, Weber L. 2015. Minor revision to V4 region SSU rRNA 806R gene primer greatly increases detection of SAR11 bacterioplankton. Aquat Microb Ecol 75:129–137. doi:10.3354/ame01753

[47] Martin M. 2011. Cutadapt removes adapter sequences from high-throughput sequencing reads. EMBnet j 17:10. doi:10.14806/ej.17.1.200

[48] Bolyen E, Rideout JR, Dillon MR, Bokulich NA, Abnet CC, Al-Ghalith GA, Alexander H, Alm EJ, Arumugam M, Asnicar F, et al.. 2019. Reproducible, interactive, scalable and extensible microbiome data science using QIIME 2. Nat Biotechnol 37:852–857. doi:10.1038/s41587-019-0209-9 31341288 PMC7015180

[49] Callahan BJ, McMurdie PJ, Rosen MJ, Han AW, Johnson AJA, Holmes SP. 2016. DADA2: high-resolution sample inference from Illumina amplicon data. Nat Methods 13:581–583. doi:10.1038/nmeth.3869 27214047 PMC4927377

[50] Wang J, Zhang Q, Wu G, Zhang C, Zhang M, Zhao L. 2018. Minimizing spurious features in 16S rRNA gene amplicon sequencing. PeerJ Preprints. doi:10.7287/peerj.preprints.26872v1

[51] Bokulich NA, Kaehler BD, Rideout JR, Dillon M, Bolyen E, Knight R, Huttley GA, Gregory Caporaso J. 2018. Optimizing taxonomic classification of marker-gene amplicon sequences with QIIME 2’s q2-feature-classifier plugin. Microbiome 6:90. doi:10.1186/s40168-018-0470-z 29773078 PMC5956843

[52] Bland JM, Altman D. 1995. Calculating correlation coefficients with repeated observations: Part 1--Correlation within subjects. BMJ 310:446. doi:10.1136/bmj.310.6977.446 7873953 PMC2548822

[53] Shi Y, Zhang L, Do K-A, Peterson CB, Jenq R. 2020. aPCoA: covariate adjusted principal coordinates analysis. Bioinformatics 36:4099–4101. doi:10.1093/bioinformatics/btaa276 32339223 PMC7332564

[54] Mallick H, Rahnavard A, McIver LJ, Ma S, Zhang Y, Nguyen LH, Tickle TL, Weingart G, Ren B, Schwager EH, Chatterjee S, Thompson KN, Wilkinson JE, Subramanian A, Lu Y, Waldron L, Paulson JN, Franzosa EA, Bravo HC, Huttenhower C. 2021. Multivariable association discovery in population-scale meta-omics studies. PLoS Comput Biol 17:e1009442. doi:10.1371/journal.pcbi.1009442 34784344 PMC8714082

